# Colposcopic magnified scanning-assisted CO_2_ laser treatment (CMSLT) for vaginal high-grade squamous intraepithelial lesion (VaHSIL): A single-center prospective cohort study

**DOI:** 10.1007/s10103-026-05095-w

**Published:** 2026-11-10

**Authors:** Annalisa Pieralli, Raffaella Venzi, Claudia Maccarrone, Claudia Giglioni, Claudia Bianchi, Giulia Fantappiè, Luca Giannoni, Irene Fusco, Massimiliano Fambrini

**Affiliations:** 1 Azienda Ospedaliero Universitaria Careggi, Firenze, Italy; 2 NOA-Nuovo Ospedale Apuane, Massa, Italy; 3 Ospedale San Giovanni di Dio Torregalli, Firenze, Italy; 4 El.En. Group, Calenzano, 50041 Italy

**Keywords:** Vaginal high-grade squamous intraepithelial lesion, CO_2_ laser, Scanner-assisted technology

## Abstract

To evaluate the feasibility of Colposcopic Magnified Scanning Laser Treatments (CMSLT), divided into laser excision (CMSLE) and vaporization (CMSLV), using a scanner-assisted CO_2_ laser as a singular outpatient therapeutic modality for patients affected by vaginal high-grade squamous intraepithelial lesion (vaHSIL). vaginal high-grade squamous intraepithelial lesion (vaHSIL) is a rare condition associated with persistent high-risk HPV infection, carrying a concrete potential for progression into invasive carcinoma. Due to diagnostic complexities and the rarity of the disease, its optimal clinical management and treatment standardization remain controversial. This prospective non-randomized cohort study was conducted between January 2022 and June 2024 on 63 patients with histologically confirmed vaHSIL. Based on the 2011 IFCPC colposcopic terminology, patients were assigned to either the CMSLV group (*n* = 22) or the CMSLE group (*n* = 41). The outpatient protocol utilized a laser system implemented with a computerized surgical scanner-assisted micromanipulator. The two-year follow-up included semi-annual screenings with HPV testing, cytology, and colposcopy. Follow-up adherence was high, with a dropout rate limited to 11.3%. The cumulative recurrence risk for vaHSIL was 14.5%, with no statistically significant difference between the two modalities, although the CMSLE group showed a higher 2-year disease-free survival rate (70% vs. 57%). The progression rate to invasive carcinoma was 1.6% 1 cases in the CMSLE cohort), revealing a 2.4% rate of occult cancer missed at initial colposcopic assignment. Only one major complication occurred (vaginal perforation, 1.6% in a post-hysterectomy patient), with no minor complications or long-term sequelae (dyspareunia or pain) reported. CMSLT may represent feasible approach since it happened to be safe, effective and associated with patient compliance. The precision of the scanner-assisted technology minimizes thermal damage and the risk of undertreating occult cancer, allowing for tailored and efficient therapy.

## Introduction

VaIN is a rare condition that precedes invasive squamous vaginal carcinoma, with an incidence rate of 0.2–0.3 cases per 100.000 women Italy [1].

VaIn represents approximately 0.4%–1%,of all intraepithelial neoplasia of the lower female genital tract.

Critical factors influencing VaIN progression and recurrence include persistent high-risk Human Papillomavirus (hrHPV) infection, advanced age (specifically over 50 years old), and Hysterectomy [2].

VaIN, like cervical intraepithelial neoplasia (CIN) is closely associated with persistent infection by high-risk human papillomavirus (HPV) genotypes, particularly HPV 16, followed by other genotypes such as 58, 73, and 31 [2–4].

Notably, hysterectomy performed for the treatment of CIN has been identified as a risk factor for subsequent VaIN occurrence, with approximately 1%–7% of patients developing VaIN months to years post-surgery [5, 6].

According to 2020 WHO terminology VaIN is histologically classified into grades based on the affected thickness of the vaginal epithelium by abnormal cells low-grade: low-grade intraepithelial lesions (vaLSIL), and high-grade squamous intraepithelial lesion (vaHSIL) [7]. vaHSIL may in some cases progress into invasive vaginal cancer. The risk of cancer ranges from 2 to 12% [1, 8, 9], and this risk increases with the duration of follow-up. There is a possibility that vaginal cancer may have been undetected at the time of initial VaIN diagnosis: the risk of occult cancer is reported to be 4.6%, and it can be as high as 10.3% in the vaHSIL cohort [10]. Due to the lack of specific symptoms, Pap smears and HPV testing and Colposcopy are crucial for early detection of VaIN. Colposcopic diagnosis of vaHSIL can be challenging due to low acetic acid reactivity of most vaginal lesion, difficulty in vascular pattern, and hiding lesions located in areas not easily to be explored [6]. Despite this inconsistency between colposcopic diagnosis and definitive histology which is usually worse than expected the 2011 International Federation for Cervical Pathology and Colposcopy (IFCPC) is still widely used to identify the risk of vaginal cancer among colposcopic Patterns (Table 1).

**Table 1:** Abnormal Colposcopic findings in 2011 IFCPC terminology of vaginal epithelium

location	upper third/lower two thirds	anterior/posterior/right/left	
Grade 1	thin aceto white epithelium	fine punctuation	fine mosaic
Grade 2	dense acetowhite epithelium	coarse punctuation	coarse mosaic
suspicious for invasion	atypical fragile vessels	necrosis/ulcer	exophitic lesion

Human Papillomavirus (HPV) vaccination is a critical primary prevention intervention, and it has proven its efficacy against cervical HPV infection, high-grade preinvasive lesions, and invasive cancers, especially when administered at an early age before sexual debut [11]. The best effectiveness of prophylactic HPV vaccination has been observed in women who had been vaccinated at an early age, since more than 70% of VaIN are associated with HPV types included in the 9-valent vaccine [3].

There is no evidence regarding the most adequate therapeutic method to be used in all cases of VaIN. However, excisional treatments such as the classically used partial or total colpectomy present important morbidity. In the last years, this circumstance has favored greater use of destructive treatments, which have fewer adverse effects and acceptable cure rates [12].

For gynecologists, the clinical approach in patients with VaIN constitutes a real challenge in health care practice. The low frequency of VaIN, the challenges associated with its diagnosis, the scarce knowledge of its natural history, and the lack of evidence and guidelines supporting a specific treatment and defining follow-up, explain the complexity in determining the most adequate clinical approach to VaIN.

This prospective cohort study was conducted to demonstrate the feasibility of Colposcopic Magnified Scanning Laser Treatments (CMSLT) as singular outpatient treatment modality for patients affected by vaHSIL. Feasibility was defined as the quality of the procedure of being safe in terms of low complications, convenient in terms of achieved clinical results and reliable as respecting patients expectations.

## Material and methods

This prospective non-randomized cohort study aimed to describe the rates of recurrence defined as histologically proven VaHSIL reappearance at scheduled follow up, progression defined as histologically proven invasive cancer at scheduled follow up, and clearance as no vaSIL, any grade, at scheduled follow up,between histologically confirmed vaHSIL colposcopically assigned to laser excision or vaporization and to compare results to common treatments described in medical literature.

**Fig. 1:**
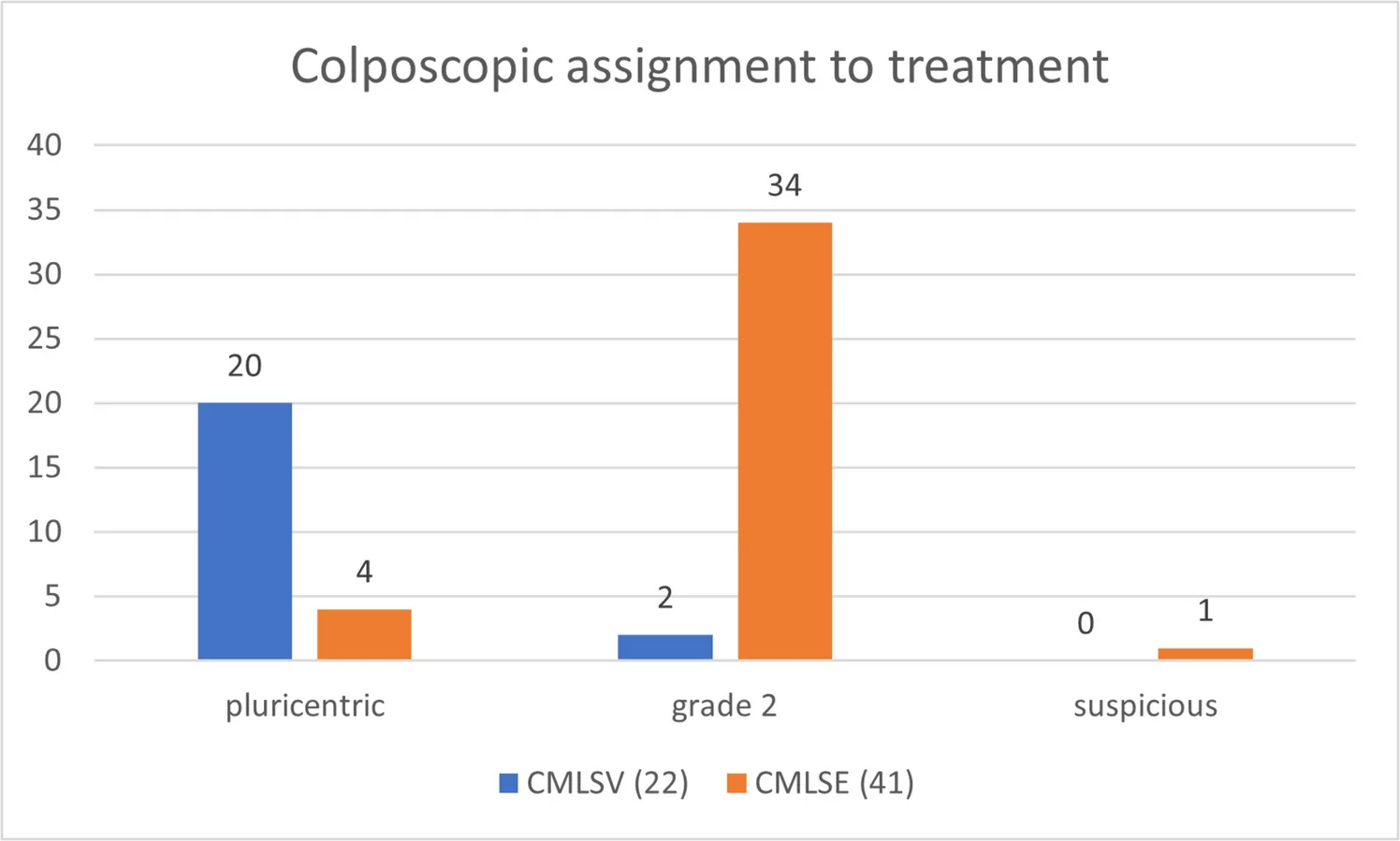
Colposcopic assigment to treatment of the the study group (lost to follow up excluded)

Patients affected by a first diagnosis histologically proven vaHSIL were recruited from January 2022 and June 2024 and colposcopically assigned into two arms: Colposcopic Magnified Scanning Laser Excision (CMSLE) group and Colposcopic Magnified Scanning Laser Vaporization (CMSLV) group. The assignment and scheduled follow up colposcopies were performed by a singular experienced colposcopist (AP, listed among Authors) according to the 2011 IFCPC Colposcopic Terminology (Table 1) and basing on two major principles: all cases of grade 1 or grade 2 of multiple lesions (> 1) were assigned to CMSLV while grade 2 on singular lesion and all cases suspicious of invasion were assigned to CMSLE. Patients affected by multiple lesions of different grades, undergoing at least 1 excision were considered in the CMSLE (Fig. 1). Dimension of the lesions was not considered as a variable and was not described.

All procedures were performed in an outpatient setting; no sedation, or general anaesthesia was administered. Local injectable anaesthetic (1:200000 Optocain® with adrenaline) was limited to wide procedures. Patients were discharged immediately after the procedure and received post-operative care instructions (no sexual intercourse, no bathing or swimming, no vaginal moisturizes or medication up to 30 days; shower allowed with common detergents; no antibiotics were prescribed; pain killers were suggested if needed according to personal allergies).

CMSLT were performed with a SmartXide CO_2_ C80H laser (DEKA M.E.L.A. Srl) in a 3D ablation mode™ at 12 watts of power in CW mode of emission and using an hexagonal scanning shape to vaporize and a rotatory 0.5 mm round shape to excise connected to a colposcope. Treatments were tailored on localization of the lesion, saving normal vaginal epithelium so to promote tissue repair. Histological analysis was performe by blinded pathologists both to treatment allocation and clinical suspicion.

All procedures were performed using a SmartXide2C80 RF-excited ultrapulsed CO_2_ laser (DEKA M.E.L.A. Srl), coupled to a scanner-assisted micromanipulator (ColpoScan, DEKA M.E.L.A. Srl) mounted on a Zeiss 150-FC colposcope equipped with a 300-mm effective focal length (EFL) lens.

ColpoScan is a micromanipulator equipped with an optical zoom based on holographic lens technology (Full Holo) and an integrated electronic scanner. This optical zoom allows the laser beam to be focused to a single spot with a diameter of 190 μm at a 300-mm EFL, enabling tissue treatment with microsurgical precision. The integrated scanner rapidly moves the focused spot across the tissue following predefined scanning patterns for excision or rapid vaporization (ablation). This configuration allows controlled energy delivery through adjustment of the laser power, dwell time, scanning pattern, and scan repetition.

Scanning patterns were used for both tissue vaporization and excision in 3D Ablation™ mode. The laser was set to continuous-wave (CW) mode at 12 W, with a scan dwell time of 0.1 ms, corresponding to a nominal fluence of 4.23 J/cm^2^ over the single focused spot. For vaporization, a filled hexagonal pattern, typically 0.9 mm wide, was used in repeated scanning mode (0.1 s pause between successive scans). The vaporization pattern size can be adjusted to the lesion dimensions, up to a maximum selectable scanning width of 4.7 mm. The system-calculated ablation depth per scan pass was approximately 0.01 mm.

For excision, the same laser emission mode, power, and dwell time were used. The hexagonal pattern was replaced with a filled circular scanning pattern 0.5 mm in diameter, and the scanner was set to continuous scanning mode (no pause between successive scans). In this cutting mode, the surgeon manually guides the scanned pattern along the intended incision line, as in conventional single-spot cutting, while the scanner controls energy distribution within the circular pattern to support cutting and coagulation.

Scanning technology improves procedural safety, reproducibility, and ease of use by allowing rapid and controlled delivery of laser energy to the tissue. It provides greater control over laser–tissue interaction, including ablation depth per scan pass and the lateral thermal effects that contribute to coagulation.

Procedures were performed after local injectable anaesthetic (1:200000 Optocain® with adrenaline). A smoke evacuator was used during the procedures for reducing smoke exposure. The aim of every laser surgical session was no residual disease. The colposcopic magnification and illumination connected to CO_2_ laser Scan-aid microspot micromanipulator helped to limit the treated areas and respect the normal tissue limiting the thermal damage.

Intraoperative haemorrhage, vaginal perforation, postoperative vaginal infection, were considered major complications; postoperative vaginal burning or dyspareunia were considered minor ones. Histological diagnosis on excised specimen.

Follow-up was considered valid if patients attended at least 4 check-up visits scheduled every 6 months (2 years) and consisted of HPV and cytology testing, as well as colposcopy with directed biopsy if needed. Patients lost at follow up were excluded. Recurrence was defined as any histologically proven vaHSIL on biopsy.

The of recurrence rate of CMSLT and the risk of undertreatment were considered primary end points.

Statistical analysis was performed using IBM SPSSc statistical software (version 28.0). Every variable was described as categorical, thus descriptive statistics were reported as absolute frequency and percentage for categorical variables. To test the difference between the two treatments to prevent a first recurrence the Fisher’s exact test was applied. The statistical significance threshold was *p* < 0.05. No statistical evaluation was performed on longitudinal recurrence.

Survival analysis was performed to evaluate time to recurrent vaSIL of any grade (relapse-free survival, RFS) across CMLST study groups and assess the need of a second treatment for patients Time-to-event was defined as the duration from baseline (t = 0) to the occurrence of a relapse event or censoring. Right-censoring was applied under two specific rules: (1) patients remaining disease-free at the end of the follow-up period, and (2) patients unable to complete the follow-up protocol or undergo evaluation due to a definitive diagnosis of cancer.

Non-parametric Kaplan–Meier survival curves were estimated for both groups, and 95% confidence intervals (CI) for the cumulative survival probabilities were computed using Greenwood’s formula with normal approximation. Standard errors (SE) were derived accordingly.

Clinical trial number: not applicable

## Results

During two years of recruitment (2022–2024) authors were sent 71 cases of vaHSIL that were eligible for the prospective study. A total of 8 patients (11.3%) were lost at follow up not providing at least 4 check-up colposcopies thus 63 patients made up the final case series: 25 patients on 63 had multiple site vaHSIL (39.7%), 35 on 63 (55.6%) had a grade 2 as preoperative IFCPC colposcopic pattern and 1 (1.6%) patient was suspected of vaginal cancer. Definitive assignment divided patients in CMSLV treated group constituted by 22 patients and CMSLE treated one constituted by 41 patients (Fig. 1). No difference among demographic characteristics was described among the two groups as shown in Table 2.

**Table 2:** Demographic, clinical, and lesion characteristics of the study population

Characteristic	Total population (*N* = 63)	Group CMLSV (*n* = 22)	Group CMLSE (*n* = 41)	*p*-value (Fisher's)
DEMOGRAPHIC AND GENERAL CHARACTERISTICS				
Average age	56.4	42.0	52.6	-
Premenopausal	25	8	17	-
Postmenopausal	38	14	24	1
Immunosuppression	3	2	1	0.282
Current smoker status	28	9	19	0.602
GYNECOLOGIC AND SURGICAL HISTORY				
Prior cervical disease CIN2 +	26	10	16	-
Previous VaIN treatment	0	0	0	-
Previous hysterectomy	29	8	21	0.288
Indication for hysterectomy				
Benign disease and CIN2 +	29	8	21	-
Malignant disease	0	0	0	-
VIROLOGICAL AND CYTOLOGICAL DATA				
high-risk genotypes hrHPV (HPV 16)	18	6	12	0.773
high-risk genotypes noHPV16	21	6	15	-
Cervical-vaginal cytology Pap smear				
Negative	0	0	0	-
LSIL—ASC-US	18	16	2	-
LSIL—ASC-US	45	17	28	-
LESION CHARACTERISTICS (IFCPC TERMINOLOGY)				
Number of lesions				
Solitary	39	2	37	-
Multiple	24	20	4	0.0001*
Lesion grade, *n* (%)				
grade 1	27	20	7	0.0001*
grade 2	36	2	34	-
Lesion location				
Upper third of vagina or vaginal vault	52	17	35	-
Middle / Lower third	11	5	6	-
Suspicion of invasion (colposcopic/clinical), n (%)	1	0	1	-

^*^ Multiple and grade 1 lesions were significantly treated by CMLSV

None of the CMSLV treated group developed vaginal cancer during the follow-up period. In contrast, among CMSLE treated patients, two cases (3.2%) of invasive disease were diagnosed representing management failure: one at the definitive histological examination following the initial treatment representing occult prevalent cancer and one at the second excision performed for a recurrent lesion detected at the 6-month follow-up. Overall, the finding of only 1 cancer after 6 months of follow up indicate a progression risk based on persistence of less than 2% (1.6%) in this case series.

All patients treated by CMSLE received intelligible histological definitive reports with no case of burned tissueVaginal specimens were not oriented, thus pathologists did not provide for margins. One case of undertreatment was identified (1/41, 2.4%), although invasive disease had been suspected at the initial colposcopic assessment.

**Fig. 2:**
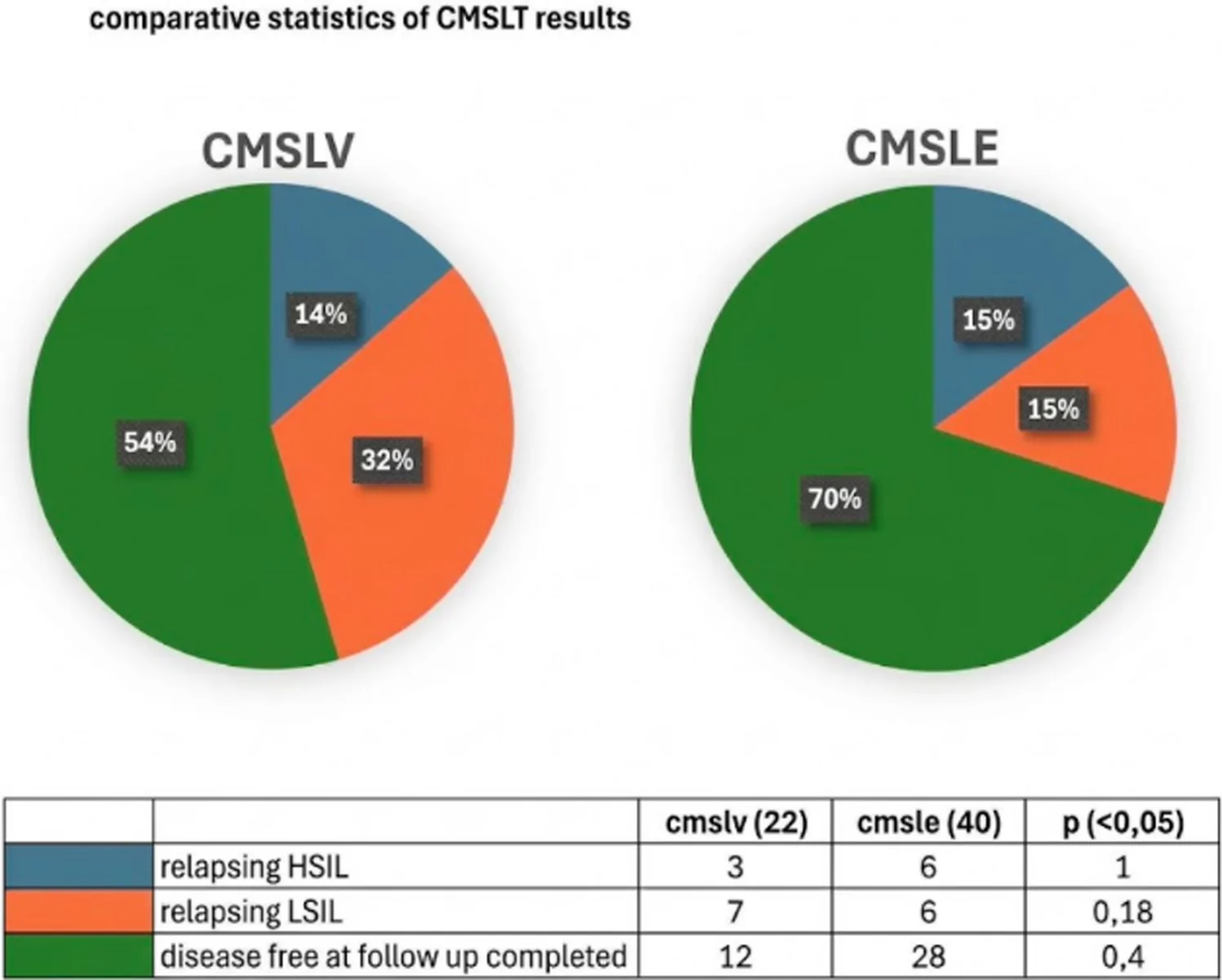
Comparative statistics of CMSLT results

Excluding the vaginal cancer diagnosed after primary CMSL T 22/62 (35.5%), patients experienced at least one histologically proven relapsing vaSIL and needed subsequent CMSLT, but only 9/62 were diagnosed as recurrent vaHSIL, thus the cumulative recurrence risk of CMSLT was 14.5% while 13/62 patients diagnosed with vaLSIL (20.5%) during follow up were not considered recurrence by definition. No statistically significant difference in term of relapsing risk was found among CLMV and CNSLE as shown in Fig. 2. Over a 24-month follow-up period, patients in the CMLSE group achieved a vaSIL recurrence-free survival rate of 85.0%, compared to 86.4% in the CMLSV group, indicating comparable disease control between the two cohorts.

One of the 63 patients (1/63, 1.6%) patient affected by vaHSIL on vaginal cuff post hysterectomy had a vaginal perforation during CMSLE that required major surgery and general anaesthesia to be sutured. No patient complained about postoperative vaginal burning or dyspareunia.

**Fig. 3:**
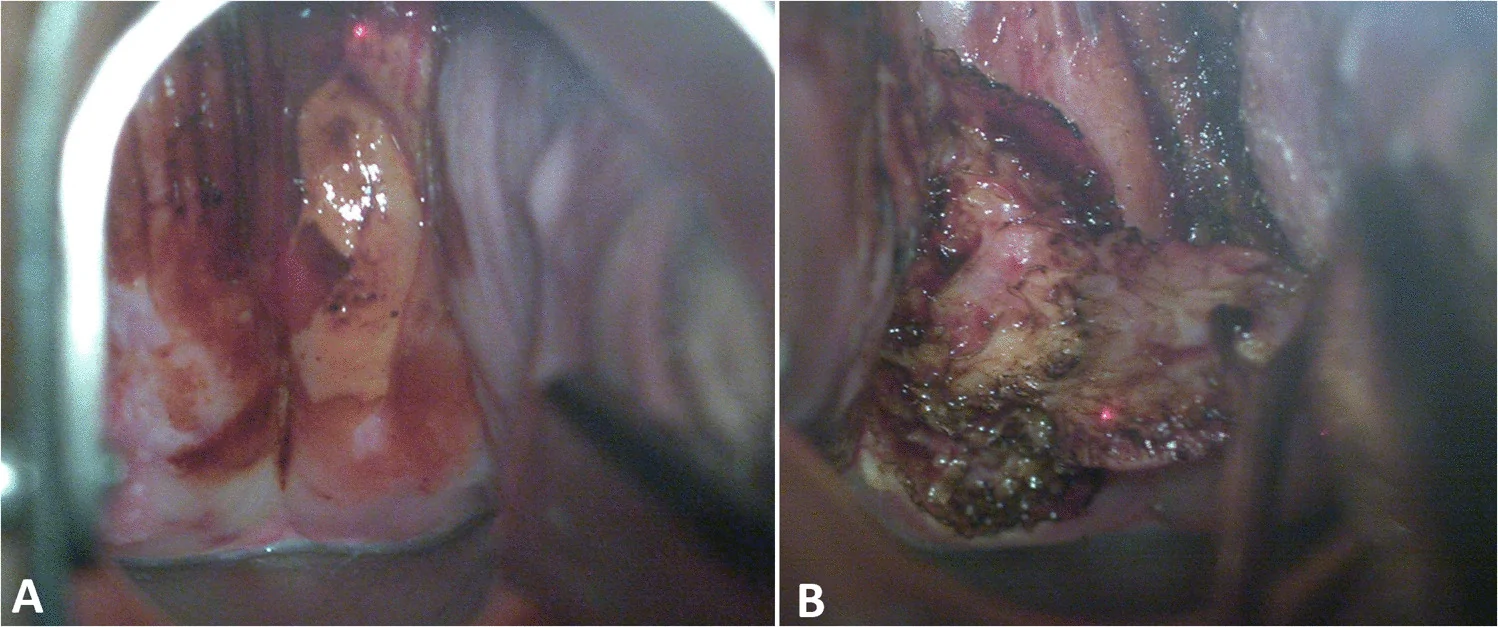
**A**;**B** Representative intraoperative images of scanner-assisted CO_2_ laser treatment for VaHSIL. Surgical field during the treatment, demonstrating targeted tissue ablation/excision achieved through ColpoScan-guided laser energy delivery while preserving adjacent healthy vaginal tissue

Representative intraoperative images of scanner-assisted CO_2_ laser treatment for VaHSIL are illustrated in Fig. 3.

## Discussion

**Fig. 4:**
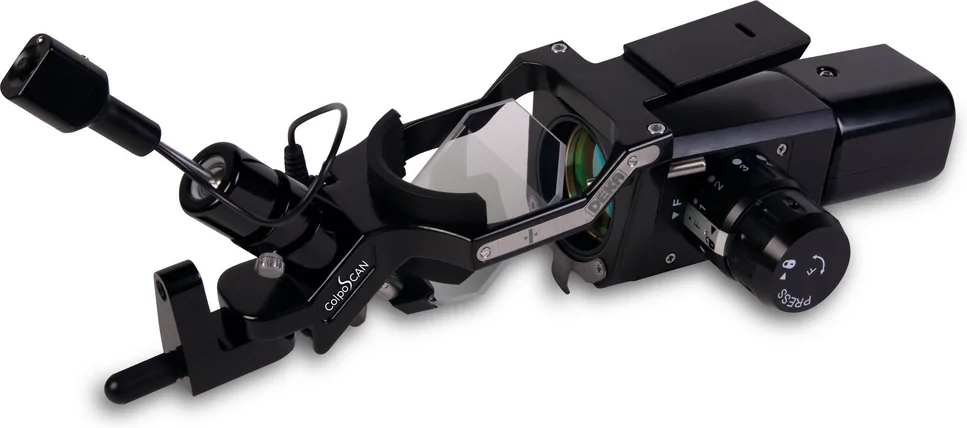
Scanning aided micromanipulator used for Colposcopic Magnified Scanning Laser Treatment (CMSLT). The system allows microsurgical laser delivery with computer-controlled scanning patterns, providing precise tissue vaporization and excision while minimizing thermal damage to surrounding tissues

The present study evaluated the feasibility, safety, and clinical outcomes of Colposcopic Magnified Scanning Laser Treatment (CMSLT), including both excision (CMSLE) and vaporization (CMSLV), performed using a scanner-assisted CO_2_ laser system (Fig. 4) for the management of vaHSIL in an outpatient setting.

The present study assessed feasibility of the procedures though three principles: safety, therapeutic convenience of the described procedure analyzed by relapsing rate and progression rate and reliability by patients’ loyalty to treatment and the adherence rate and duration of follow-up.

In studies dedicated to pre-invasive vaginal lesions dropout or non-adherence rates vary significantly depending on the time window analyzed: initial adherence is generally high. In clinical trials, the loss to follow-up at six months hovers around 15–18% [13, 14]. The most critical drop-off period was registered at 12 months after the treatment; studies focusing on the follow-up of pre-neoplastic lesions show that Loss to Follow-Up (LTFU) rates can range from 30% to dramatic peaks of 65% at 12 months post-treatment, particularly in settings with less integrated screening programs or in the absence of active patient recall systems [12–14].

In the present study authors registered only a 11% drop out rate, despite a 35.5% risk of relapse and consequent re-treatment which underlines patients' trust and loyalty to this procedure. This result confirms previous literature data, which shows that patients undergoing topically administered medical therapies (e.g., Imiquimod) have lower long-term adherence compared to those who undergo surgical excision or laser treatment vaporization, likely due to a different perception of the disease’s severity [15, 16].

Since the high relapsing nature of the disease paired to its frequent multifocal aspect, The International Consensus Statements (ESGO/ISSVD/ECSVD/EFC) suggest follow up to be scheduled as soon as 6 months after the first treatment and to be repeated regularly for the first 2 years, transitioning to an annual basis thereafter. Respecting these guidelines is thus strictly dependent on patients’ loyalty to treatment which is essential to assure long-term post treatment follow-up [18–20].

Across retrospective studies and clinical series, carbon dioxide (CO_2_) laser therapy was generally classified as a safe, low-impact, and minimally invasive treatment modality [18–21].

The overall reported complication rate ranged between 5 and 15%, with the vast majority being classified as minor. The literature reported that laser excisions for high-grade VaIN found an overall complication rate of 7.8%. Of these, 90% were minor, and only 0.8% resulted in a major complication. Postoperative Bleeding was reported in roughly 2% to 7% of cases. It can occur intraoperatively, within hours of the procedure, or a few weeks later when the thermal eschar (scab) sloughs off. Most cases were controlled with local hemostatic agents, vaginal packing, or occasionally a minor hemostatic suture. Local Pain and Discomfort: highly common but transient, secondary to the raw mucosal surface and thermal energy. Literature noted it typically resolves within 2 to 4 weeks as re-epithelialization completes. Infection: documented in approximately 1% to 4% of patients. Vaginal cuff or localized wall infections were managed effectively with standard topical or oral antimicrobial therapies. Pelvic Organ Perforation (Extremely Rare): because the vaginal wall is thin (1.5 mm to 3 mm) and adjacent to the bladder and rectum, excessive depth of laser penetration can cause perforation. In major surgical series, the rate of vaginal vault perforation or fistula formation is < 1% [18–20].

Chronic Long-Term Complications are described too such as Vaginal Stenosis and Synechiae (Adhesions) occurring in 3% to 12% of patients or Dyspareunia as a direct consequence of vaginal stenosis, loss of mucosal elasticity, or extensive scarring.

To keep complication rates at the lower end of the statistical spectrum, the literature heavily recommends: limiting vaporization depth strictly to 1 mm to 1.5 mm (to clear the epithelium and lamina propria without penetrating deeper muscular layers), utilizing pre- and post-operative topical estrogen therapy in postmenopausal women to optimize tissue thickness and healing, incorporating postoperative vaginal dilators, once healed, to prevent the formation of synechiae/adhesions [18, 19]. Limiting vaporization depth or normalizing thickness of excision for the entire lesion was generally challenging with common laser devices and it required high surgical skills that can be reached out only after a long learning curve, thereby limiting the technique spread.

This may explain why the international literature has identified vaginally administered 5% imiquimod as the most reliable treatment option for for vaSIL. Nonetheless it was associated with significant local inflammatory responses. Since Imiquimod is an immunomodulatory agent, local inflammation constitutes part of its mechanism of action; local side effect reaches 60% of the cases. These are the reasons why temporary treatment interruption occurs between 20 to 30% and definitive discontinuation rate can reach 10% rising relapsing rate [15–17].

No adverse events or complications, including hemorrhage and infection, were observed, and no postoperative complaints of pain or dyspareunia were reported. These findings represented a major strength of CMLST. These results were directly attributable to the scanner-assisted technique, which allowed surgeons to minimize treated areas, easily control depth of vaporization and excision and reduce the thermal spread. These characteristics were fundamental in treating lesions occurring after Hysterectomy. Treating the apex carried a slightly higher risk of major complications (like vault perforation) due to the proximity of the bladder and bowel loops tied down by post-surgical adhesions. Prior pelvic radiation leads to a compromised, hypoxic, and poorly vascularized vaginal tissue with a risk of non-healing ulcers and tissue necrosis after laser-treatments. The present study showed no thermal long-term complications but still 1 case of vault perforation, underlining the extreme challenge in treating this localization although performing with a computerized scanning implemented laser emission device [18–22].

Moreover, CMLST allowed authors to obtain a definitive histological result on patients who underwent excision, which allowed them to underline that colposcopically-guided procedures were essential to eliminate overtreatment and to select the appropriate laser treatment modality, thereby reducing undertreatment of a 2.4% of hidden vaginal cancer [23, 24].

## Limitations

The present study was principally limited by the small sample size of the case series and by the short time of follow up which may influence the statistical results. Moreover since Treatment allocation was based on colposcopic aspect and suspicion of invasion made by expertized surgeon (AP), Authors are aware that this is a principle confounding factor of direct comparisons between CMSLE and CMSLV in different condition and may be a limitation to reproducibility in other settings.

The abscennce of a conventional CO_2_ laser control group denies the possibility of defining superiority of scannered based procedures, however the study was primarily intended to be descriptive, with the aim of expanding current knowledge on the advantages offered by the application of this novel technology to conventional laser treatments, although. Furthermore, it clarified that colposcopic guidance in vaginal pathology was not only fundamental at the time of the diagnosis but at the time of treatment modality assignment and procedure too.

## Conclusions

Despite increasing interest in vaSIL, the progression rate and optimal management strategies remain uncertain and highly controversial. Management requires individualization based on patient characteristics and disease extension. While vaLSIL often regress spontaneously and may be managed by observation, vaHSIL typically require active intervention due to their premalignant potential. Treatment modalities include surgical excision, laser ablation, and topical agents like Imiquimod as shown in guidelines without describing real characteristics of them and safety or reliability.

CO_2_ laser surgery was already considered without scanning aided micrometric spot micromanipulators an effective therapeutic option because it provides adequate oncological management with good functional results, but it was considered challenging for surgeons and risky for patients thus not to be proposed on a large scale. CMLST instead allows maintenance of a uniform depth throughout the sample during the excision and the ablation. CMLST adjusts the treatment to the size of the lesion, minimizing thermal damage and controlling bleeding. CMLST offers to surgeons the possibility to easily switch from ablation to excision modality customizing the treatment onto colposcopic diagnosis and ensuring margin precision both in surface and in depth.

## Data Availability

The data that support the findings of this study are not publicly available since it contains information that could compromise the privacy of research participants, but are available from the corresponding author [IF] upon reasonable request.
